# Hypothesis testing of meiotic recombination rates from population genetic data

**DOI:** 10.1186/s12863-014-0122-7

**Published:** 2014-11-30

**Authors:** Junming Yin

**Affiliations:** Department of Management Information Systems, Eller College of Management, University of Arizona, Tucson, 85721 USA

**Keywords:** Recombination rates, Gene conversion, Hypothesis testing

## Abstract

**Background:**

Meiotic recombination, one of the central biological processes studied in population genetics, comes in two known forms: crossovers and gene conversions. A number of previous studies have shown that when one of these two events is nonexistent in the genealogical model, the point estimation of the corresponding recombination rate by population genetic methods tends to be inflated. Therefore, it has become necessary to obtain statistical evidence from population genetic data about whether one of the two recombination events is absent.

**Results:**

In this paper, we formulate this problem in a hypothesis testing framework and devise a testing procedure based on the likelihood ratio test (LRT). However, because the null value (i.e., zero) lies on the boundary of the parameter space, the regularity conditions for the large‐sample approximation to the distribution of the LRT statistic do not apply. In turn, the standard chi‐squared approximation is inaccurate. To address this critical issue, we propose a parametric bootstrap procedure to obtain an approximate *p*‐value for the observed test statistic. Coalescent simulations are conducted to show that our approach yields accurate null *p*‐values that closely follow the theoretical prediction while the estimated alternative *p*‐values tend to concentrate closer to zero. Finally, the method is demonstrated on a real biological data set from the telomere of the *X* chromosome of African *Drosophila melanogaster*.

**Conclusions:**

Our methodology provides a necessary complement to the existing procedures of estimating meiotic recombination rates from population genetic data.

**Electronic supplementary material:**

The online version of this article (doi:10.1186/s12863-014-0122-7) contains supplementary material, which is available to authorized users.

## Background

Meiotic recombination is one of the essential evolutionary factors responsible for promoting genetic diversity within species. There are two major types of meiotic recombination events: crossovers and gene conversions. Unlike crossover, which is a reciprocal event, gene conversion is a unidirectional event that involves the transfer of a short segment of one parental chromosome (called a ‘conversion tract’) to the other parental chromosome. Crossovers and gene conversions play different roles in shaping the pattern of linkage disequilibrium (LD) observed in natural populations: “Recombination between pairs of markers that are far apart are almost exclusively crossovers, whereas pairs of markers that are close together are affected by both crossovers and gene conversion events” [1]. Thus, studying these two biological processes and characterizing their basic parameters has direct implications for population genetic studies.

There is a growing body of work on coalescent‐based statistical approaches to jointly estimating the crossover rate, the gene conversion rate, and the mean conversion tract length from population genetic data. Building on a popular framework called the “Product of Approximate Conditionals” (PAC) model [2], Gay et al. [3] have proposed a likelihood‐based method to incorporate gene conversion events. Yin et al. [4] have extended and improved the model further by explicitly modeling overlapping gene conversion events. On the flip side of these two frequentist approaches, Bayesian Markov chain Monte Carlo (MCMC) techniques have also been developed to estimate recombination rates from population genetic data [5,6].

Despite the marked progress in the joint estimation of the aforementioned three parameters, these methods are less suitable when one of the two recombination events is absent in the genealogical model. The corresponding population parameter, especially the gene conversion rate when the gene conversion event is nonexistent, tends to be overestimated by the maximum likelihood (or maximum a posteriori) point estimation. This is unfortunately inevitable because the true parameter value (i.e., zero) lies on the boundary of the parameter space. The use of inaccurate parameters may limit the efficacy of these approaches, and can also hinder population genetic analyses based on these estimators. Therefore, it has become necessary to obtain statistical evidence from population genetic data about whether one of the two recombination events is absent.

The goal of this article is to propose a rigorous procedure to perform hypothesis testing for this problem. Our approach is based on the likelihood ratio test (LRT). One of the classical regularity conditions for the asymptotic distribution of the LRT statistic requires the null value to be an interior point in the parameter space. However, because this condition is not satisfied, it is invalid to apply the standard chi‐squared approximation in this setting. We thus develop a parametric bootstrap procedure to obtain an approximate *p*‐value of the observed test statistic. Coalescent‐based simulations are conducted to demonstrate the soundness and effectiveness of our approach. The bootstrap estimates of the null p‐values closely follow the theoretical prediction, while the estimated alternative *p*‐values tend to concentrate closer to zero. Finally, we apply the method to a real biological data set from the telomere of the *X* chromosome of African *D. melanogaster*. The result suggests that while gene conversion is likely to play a leading role in shaping the observed polymorphism in these regions, crossover may not have been greatly suppressed in a short segment of *su*(*w*^*a*^) locus.

## Methods

We begin by reviewing some previous statistical models used for *point estimation* of recombination parameters from population genetic data. In developing our *hypothesis testing* procedure based on the likelihood ratio test (LRT), we adopt the likelihood function of the OVERPAINT model that offers greater flexibility by allowing for overlapping gene conversions [4,7]. Throughout this paper, *ρ* and *γ* are used to refer to the population‐scaled crossover and gene conversion rates (per kb), respectively. The mean length of gene conversion tracts (kb) is denoted by *λ*.

### The PAC model and the GenCo model

In principle, given a set of *n* haplotypes *H*={*h*_1_,…,*h*_*n*_} sampled from a natural population, the estimation of *ρ*,*γ* and *λ* can be obtained by maximizing the likelihood function $\ell (\rho, \gamma, \lambda) := \mathbb {P}(H \mid \rho, \gamma, \lambda)$. However, unless we can examine the true genealogical history of sampled sequences in the population [8], which is rarely available in a population genetic study, we are unable to compute the exact likelihood function in most models of interest. To be precise,

$$ \ell(\rho, \gamma, \lambda)\!:=\! \mathbb{P}(H \!\mid\! \rho, \gamma, \lambda)= \int \mathbb{P}(H \mid G)\, \mathbb{P}(G\mid \rho, \gamma, \lambda) \, dG, $$

where the integral is over all possible genealogies *G* and $\mathbb {P}(G\mid \rho, \gamma, \lambda)$ is modeled by the coalescent process with crossovers and gene conversions [9,10]. The above likelihood computation is notoriously difficult because the number of genealogies *G* consistent with the sampled haplotypes *H*, where the consistency is determined by $\mathbb {P}(H \mid G)$, grows extremely fast as the length of sampled haplotypes increases [11]. Several approximate‐likelihood approaches have therefore been developed to approximate the likelihood surface. The ‘Product of Approximate Conditionals’ (PAC) model, first proposed in [2], makes use of the fact that the joint likelihood of the sampled haplotypes can be decomposed into a product of conditional probabilities:

$$\begin{aligned} \ell(\rho, \gamma, \lambda) & := \mathbb{P}(h_{1}, \ldots, h_{n} \mid \rho, \gamma, \lambda) = \mathbb{P}(h_{1}\mid\rho, \gamma, \lambda) \\ & \quad\times \mathbb{P}(h_{2} \mid h_{1}, \rho, \gamma, \lambda) \times \cdots \\ &\quad\times \mathbb{P}(h_{n} \mid h_{1}, \ldots, h_{n-1}, \rho, \gamma, \lambda).  \end{aligned} $$

However, the exact conditional probabilities $\mathbb {P}(h_{k+1} \mid h_{1}, \ldots, h_{k}, \rho, \gamma, \lambda)$ are largely unknown for the coalescent models with recombination. Using efficiently computable approximations $\hat {\pi }$ to substitute for the exact conditional probabilities , the following approximation to the joint likelihood has been suggested in [2]:

$$\begin{aligned} \ell(\rho, \gamma, \lambda) & \approx \ell_{\textrm{PAC}}(\rho, \gamma, \lambda) = \hat{\pi}(h_{1}\mid\rho, \gamma, \lambda) \\ &\quad \times \hat{\pi}(h_{2} \mid h_{1}, \rho, \gamma, \lambda) \times \cdots\\ &\quad \times \hat{\pi}(h_{n} \mid h_{1}, \ldots, h_{n-1}, \rho, \gamma, \lambda).  \end{aligned} $$

Instead of maximizing the true but intractable likelihood function *ℓ*, the idea of the PAC model is to use the approximate likelihood *ℓ*_PAC_ as a surrogate function to estimate recombination parameters from the sampled haplotypes. The original PAC model [2] has only considered the estimation of the crossover rate *ρ*, in which case *ℓ*_PAC_ becomes a one dimensional function. Gay et al. [3] have extended the model by incorporating gene conversion events, and their model GenCo can be used to jointly estimate the crossover rate *ρ*, the gene conversion rate *γ*, and the mean conversion tract *λ*.

The choice of the approximate conditional probabilities $\hat {\pi }(h_{k+1} \mid h_{1}, \ldots, h_{k}, \rho, \gamma, \lambda)$ in the GenCo model assumes that *h*_*k*+1_ is an imperfect mosaic copy of *h*_1_,…,*h*_*k*_. In particular, *h*_*k*+1_ is considered to consist of a mixture of segments from *h*_1_,…,*h*_*k*_ with a small number of mutations, and its mosaic structure is the result of a joint effort by the crossover and gene conversion events. To capture this imperfect copying process, Gay et al. [3] have designed a factorial hidden Markov model (HMM) [12,13] with two independent hidden chains. The crossover chain is modeled as a Poisson process with rate *ρ* along the sequence; for the gene conversion chain, both initiation and termination of a conversion tract are modeled as Poisson processes, with rates *γ* and 1/*λ* respectively. The joint configuration of the states in these two chains determines the index of the haplotype from which the copying is performed. See [3] and Figure two(a) in [4] for more details.

### The OVERPAINT model

**Figure 1:**
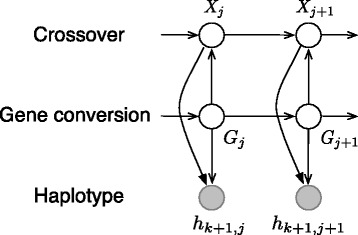
**Interleaved HMM.** The interleaved HMM with coupled hidden chains used in the OVERPAINT model to compute $\hat {\pi }(h_{k+1} \mid \, h_{1}, \ldots, h_{k}, \rho, \gamma, \lambda)$ [adapted from Figure two(b) of [4]]. *h*_*k*+1,*j*_ is the allele state at the *j*‐th site of haplotype *h*_*k*+1_. *X*_*j*_ and *G*_*j*_ denote the *j*‐th hidden state of the crossover and gene conversion chain, respectively, and their joint configuration determines the index of the haplotype from which *h*_*k*+1,*j*_ is copied.

Because gene conversion events involve non‐reciprocal transfer of genetic information between homologous sequences, the typical product created by a gene conversion event is a descendant sequence that consists of a prefix of a sequence *h* followed by a short internal fragment of another sequence *h*^′^, which is then followed by a suffix of the first sequence *h*. However, the independent assumption of the two hidden chains in the factorial HMM formulation of the GenCo model cannot capture this alternating pattern of the descendant sequence. An improved model called OVERPAINT based on an interleaved HMM (Figure 1) is introduced in [4]. The desired alternating pattern is achieved by coupling the crossover and gene conversion chains as well as by defining their new transition probabilities. In Figure 1, direct edges from the gene conversion chain to the crossover chain constrain the crossover chain to stay in the same state as the previous site whenever the current site is in a gene conversion tract. To be precise, the transition probability of the crossover chain is specified as

$$  \mathbb{P}\left(X_{j+1} \mid X_{j}, G_{j+1}\right) = \left\{ \begin{array}{ll} \mathbb{P}\left(X_{j+1} \mid X_{j}\right), & \;\;\text{if}\ \ G_{j+1} = \emptyset,\\ \mathbb{I}\left(X_{j+1} = X_{j}\right), & \;\;\text{if}\ \ G_{j+1} \neq \emptyset. \end{array} \right. $$

If site *j*+1 is not in a conversion tract (*G*_*j*+1_ is in the null state *∅*), the crossover chain evolves according to the same Poisson process as defined in the GenCo model [3]. Otherwise, if site *j*+1 is in a conversion tract (*G*_*j*+1_≠*∅*), the crossover chain keeps track of the state in the previous site, i.e., the indicator function  sets *X*_*j*+1_=*X*_*j*_.

In addition to constructing coupled hidden chains to capture the alternating pattern of gene conversion, another key feature of the OVERPAINT model is to allow for *overlapping* gene conversion events in the copying process. This is motivated by the observation that it is possible for the coalescent model with gene conversion to generate genealogies in which the gene conversion tracts partially overlap or are completely nested within each other. See [4] and [7] for details of the OVERPAINT model, including the exact form of the initial and transition probabilities of hidden chains as well as the forward‐backward algorithm to compute the approximate conditional probabilities $\hat {\pi }(h_{k+1} \mid h_{1}, \ldots, h_{k}, \rho, \gamma, \lambda)$.

Finally, by taking into account the prior information that the tract length typically ranges between 0.05 and 2 kb [14,15], a prior on the mean tract length *λ* can be imposed:

(1) $$  \log_{10}(\lambda) \sim N(-0.5, 0.4^{2}),  $$

where *N*(*μ*,*σ*^2^) denotes a standard normal distribution with mean *μ* and variance *σ*^2^. This prior is deliberately chosen to ensure $\mathbb {P}(\lambda \in [0.05, 2]) = 95\%$. A standard derivative‐free optimization algorithm, the Nelder‐Mead simplex‐reflection method [16], is applied to find the best point estimates of *ρ*,*γ*,*λ* by maximizing the posterior

(2) $$  L_{\textsc{OVERPAINT}}(\rho, \gamma, \lambda \mid H) \propto f(\lambda) \times \ell_{\textsc{OVERPAINT}}(\rho, \gamma, \lambda).  $$

Here, we use *ℓ*_OVERPAINT_(*ρ*,*γ*,*λ*) to refer to the likelihood function of the OVERPAINT model and *f*(*λ*) to denote the density of *λ* that corresponds to (1). The prior can also be interpreted as a regularizer to penalize very small or very large values of *λ*, and hence can yield more stable numerical results [7].

### Motivation examples

In the settings of nonzero crossover and nonzero gene conversion rates, the studies in [4,7] have shown that the OVERPAINT model provides a substantial improvement over the GenCo model in the accuracy of point estimation. However, as we will show below, the point estimators tend to be inflated and thus become unreliable when one of the recombination rates lies on the boundary of the parameter space, i.e., *ρ*=0 or *γ*=0. In conducting the simulation, 100 data sets with gene conversions only (*ρ*=0) and crossovers only (*γ*=0 and *λ*=0), respectively, are independently generated by the coalescent simulation program MS [17]. In each simulation, we generate a 20 kb region using *θ*=1.0/kb for the mutation rate and *λ*=0.5 kb for the mean tract length if the gene conversion rate *γ*≠0, then we obtain the point estimation of all three parameters *ρ*,*γ* and *λ* by maximizing (2).

Table 1 summarizes the parameter estimates on the data sets generated with gene conversions only (i.e., the crossover rate *ρ*=0). The column labeled $\hat \rho $ displays the mean and standard deviation (shown in parentheses) of the estimates of *ρ*. It indicates that the estimates of *ρ* are well behaved over a range of simulated data sets with gene conversion rate *γ*=0.5,1.0,2.5,5.0,10.0/kb, though they are slightly biased upward on the data sets simulated with a large gene conversion rate (*γ*=10.0/kb). In contrast, as the column labeled $\hat \gamma $ of Table 2 shows, the estimates of *γ* are significantly inflated when there is actually no gene conversion (i.e., *γ*=0). Gay et al. [3] have made the same observation about an overestimation of the gene conversion rate *γ* by their model GenCo, when gene conversion is nonexistent (see their Figure three).

**Table 1:** **Summary of parameter estimates on simulated data sets with gene conversions only (** ***ρ*** **=0)**

***γ***	$\hat {\rho }$ ^**a**^	$\hat {\gamma }$ ^**a**^	$\hat {\lambda }$ ^**a**^	$\#(\hat {\rho }; {0.05})$ ^**b**^	$\#(\hat {\rho }; {0.1})$ ^**b**^
0.5	0.03(0.05)	1.50(1.21)	0.56(0.23)	60	74
1.0	0.03(0.05)	1.81(2.01)	0.59(0.22)	77	90
2.5	0.05(0.06)	3.08(1.77)	0.54(0.19)	90	99
5.0	0.05(0.07)	4.55(1.69)	0.52(0.14)	96	99
10.0	0.12(0.15)	9.31(4.18)	0.48(0.15)	97	100

For each value of the gene conversion rate ***γ*** (per kb), 100 data sets with a sample size *n* = 20 are independently generated using the MS program [17] with a mutation rate ***θ***= 1.0/kb and a mean tract length ***λ***= 0.5 kb.

^a^The mean and SD (in parenthesis) of the parameter estimates.

^b^#$(\boldsymbol {\hat {\rho }}; \textit {k})$: the number of data sets with $\boldsymbol {\hat {\rho }}$ in the range (0,*k* ***γ***).

**Table 2:** **Summary of parameter estimates on simulated data sets with crossovers only (** ***γ*** **=0)**

***ρ***	$\hat {\rho }$ ^**a**^	$\hat \gamma $ ^**a**^	$\hat {\lambda }$ ^**a**^	$\#(\hat {\gamma }; {0.05})$ ^**b**^	$\#(\hat {\gamma }; {0.1})$ ^**b**^
0.5	0.45(0.22)	0.71(0.62)	0.66(0.25)	6	11
1.0	0.75(0.29)	0.71(0.60)	0.73(0.28)	4	10
2.5	1.54(0.68)	0.78(0.61)	0.81(0.25)	14	19
5.0	2.59(0.96)	1.21(0.79)	0.79(0.22)	7	20
10.0	5.24(8.94)	2.89(2.81)	0.75(0.29)	4	13

For each value of the crossover rate ***ρ*** (per kb), 100 data sets with a sample size *n* = 20 are independently generated using the MS program [17] with a mutation rate ***θ***= 1.0/kb.

^a^The mean and SD (in parenthesis) of the parameter estimates.

^b^#$\boldsymbol {\hat {\gamma }}; \textit {k}$ : the number of data sets with $\hat \gamma $ in the range (0,*k* ***ρ***).

In what follows, we will mainly focus on testing the null hypothesis *H*_0_:*γ*=0 (no gene conversion), but our testing procedure as outlined in Algorithm 1 can also be easily modified to testing *H*_0_:*ρ*=0, as we will demonstrate in the section of “Results and discussion”.

### Parametric bootstrap

It seems inevitable to obtain an overestimation of the gene conversion rate when *γ*=0 because the true value lies on the boundary of the possible range. We formulate and address this problem in a hypothesis testing framework, and devise a testing procedure based on the likelihood ratio test (LRT). Our null hypothesis is *H*_0_:*γ*=0 (no gene conversion), and the test statistic of the sampled haplotypes *H* is the likelihood ratio statistic:

(3) $$  \Lambda(H) = -2\log\left\{ \frac{\sup_{\rho} L_{\textsc{OVERPAINT}}(\rho, 0,0\mid H)}{\sup_{\rho, \gamma,\lambda} L_{\textsc{OVERPAINT}}(\rho, \gamma,\lambda\mid H)} \right\},  $$

where *L*_OVERPAINT_(*ρ*,0,0∣*H*) denotes the function in (2) computed with crossover rate *ρ* only (i.e., the original PAC model in [2]).

**Figure 2:**
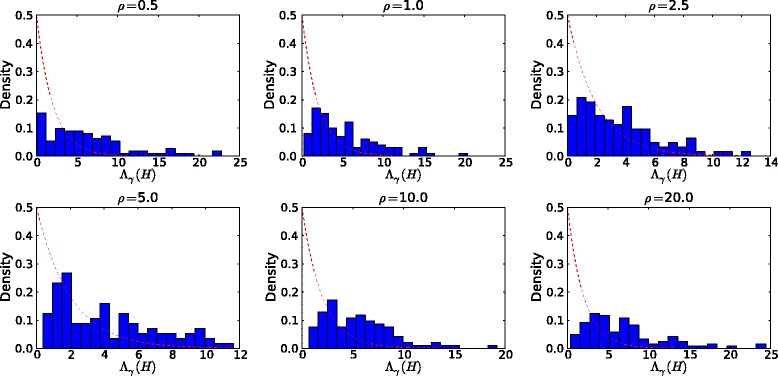
**Histograms of the LRT statistic** ***Λ*** **(** ***H*** **) under the null hypothesis** ***H***_**0**_ **:** ***γ*** **=0 (** ***n*** **=35).** For each value of the nuisance parameter *ρ* (per kb), 100 data sets with a sample size of *n*=35 are independently generated using the MS program [17] with a mutation rate *θ*=1.0/kb. The 95*%* quantiles of the histograms are: 16.99 (*ρ*=0.5), 14.36 (*ρ*=1.0), 8.32 (*ρ*=2.5), 9.73 (*ρ*=5.0), 13.04 (*ρ*=10.0), and 17.17 (*ρ*=20.0), respectively. The red dashed lines correspond to the density of ${\chi _{2}^{2}}$ distribution.



As usual, large values of the observed statistic *Λ*(*H*) would lead us to favor the alternative hypothesis and possibly to reject the null hypothesis *H*_0_. The key question is: what is the critical value of *Λ*(*H*) used to reject *H*_0_? One might conjecture that the LRT statistic in (3) would follow an asymptotic ${\chi _{2}^{2}}$ distribution under the null hypothesis. However, as Figure 2 and Additional file 1: Figure S1 show, the null distribution of the LRT statistic *Λ*(*H*) is not well approximated by the desired ${\chi _{2}^{2}}$ distribution, as least not for a sample size of *n*=35. Even for larger sample sizes, we believe that the chi‐squared approximation is still inaccurate because of two facts: first, the null value lies on the boundary of the parameter space; second, the model is not identifiable, i.e., two distinct parameter settings *γ*=0 and *λ*=0 give rise to the same likelihood. Therefore, the regularity conditions of the classical large‐sample theory are violated, and it becomes invalid to apply the standard large‐sample approximation to the distribution of the LRT statistic *Λ*(*H*) [18].

As Figure 2 and Additional file 1: Figure S1 show, the null distribution of the LRT statistic *Λ*(*H*) and its critical value (the 95*%* quantile) depends on the crossover rate *ρ*, which is an unknown *nuisance parameter* under the null hypothesis *H*_0_. This observation motivates us to develop a parametric bootstrap procedure [19] to obtain an approximate *p*‐value for the observed test statistic *Λ*(*H*), as outlined in Algorithm 1. Instead of constructing the whole null distribution of the LRT statistic, we draw *B* samples of size *n* from the null hypothesis with a crossover rate of $\hat {\rho }$, which is the parametric estimate of the nuisance parameter *ρ* under *H*_0_. We then evaluate the test statistic on each bootstrap sample, and count the proportion that exceed the observed statistic.

## Results and discussion

### Simulation study

To evaluate the performance of our testing procedure, we use the same parameter settings as in the section “Motivation examples” to conduct the simulation. All reported *p*‐values are based on *B*=200 bootstrap samples.

#### *p*‐values under the null hypothesis

**Figure 3:**
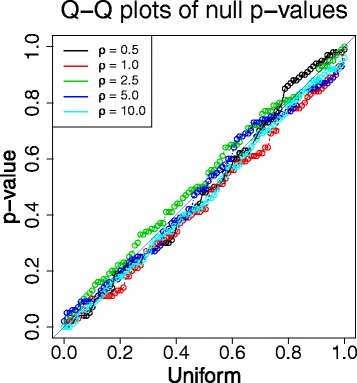
**Bootstrap estimates of the** ***p*** **‐values under the null hypothesis** ***H***_**0**_ **:** ***γ*** **=0 (** ***n*** **=35).** For each value of the crossover rate *ρ* (per kb), 100 data sets with a sample size of *n*=35 are independently generated using the MS program [17] with a mutation rate *θ*=1.0/kb. Shown in the figure are the Q‐Q plots of the *p*‐values estimated by *B*=200 parametric bootstrap replications versus a uniform distribution.

Under the null hypothesis *H*_0_:*γ*=0, we use the values 0.5,1.0,2.5,5.0 and 10.0/kb for the crossover rate *ρ* (the nuisance parameter). For each value of *ρ*, we generate 100 simulated data sets with sample sizes of *n*=20 and *n*=35 haplotypes, respectively. We then apply our parametric bootstrap procedure presented in Algorithm 1 to compute an estimate of the *p*‐value for each data set. Figure 3 and Additional file 1: Figure S2 show that the bootstrap estimates of the null *p*‐values closely follow the uniform distribution over the interval (0,1), thereby exhibiting excellent agreement with theoretical prediction. Table 3 summarizes the estimated nuisance parameter *ρ* under the null hypothesis (line 3 in Algorithm 1) that are used to draw bootstrap replications (line 4 in Algorithm 1). Though the estimates are slightly biased downwards for large values of true *ρ*, the empirical behavior shown in Figure 3 and Additional file 1: Figure S2 suggests that it suffices to draw bootstrap samples from approximately correct null distributions in our case to obtain good estimates of the null *p*‐values.

**Table 3:** **Summary of the estimated nuisance parameter** ***ρ*** **under the null hypothesis** ***H***_**0**_ **:** ***γ*** **=0**

***ρ***	***n*** **=20**			***n*** **=35**		
	$\hat {\rho }$ ^**a**^	$\#(\hat {\rho }; {2})$ ^**b**^	$\#(\hat {\rho }; {5})$ ^**b**^	$\hat {\rho }$ ^**a**^	$\#(\hat {\rho }; {2})$ ^**b**^	$\#(\hat {\rho }; {5})$ ^**b**^
0.5	0.65(0.26)	87	100	0.71(0.22)	91	100
1.0	1.04(0.37)	94	100	1.09(0.31)	99	100
2.5	2.00(0.58)	89	100	2.22(0.47)	99	100
5.0	3.33(0.75)	90	100	3.72(0.64)	97	100
10.0	7.52(1.40)	75	100	8.19(1.01)	88	100

These estimates, computed as $\boldsymbol {\hat {\rho }} = \text {argmax}_{\boldsymbol {\rho }} \textit {L}_{\textsc {OVERPAINT}}(\boldsymbol {\rho }, \text {0}, \text {0})$, are used to draw bootstrap replications (line 4 in Algorithm 1) and then to estimate the bootstrap *p*‐values (as in Figure 3 and Additional file 2: Figure S2).

^a^The mean and SD (in parenthesis) of the estimates of ***ρ***.

^b^#$\boldsymbol {\hat {\rho }}; \textit {k}$: the number of data sets with $\boldsymbol {\hat {\rho }}$ within a factor of *k* from the true ***ρ***.

#### *p*‐values under the alternative hypothesis

**Figure 4:**
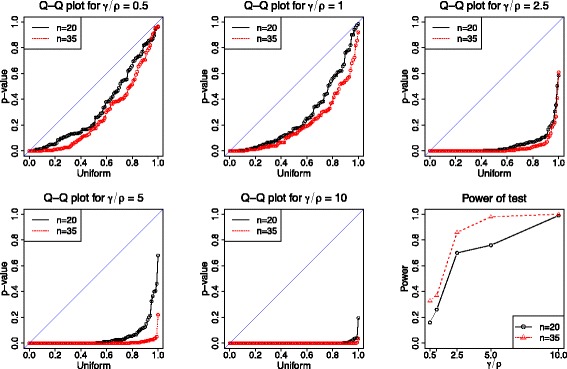
**Bootstrap estimates of the** ***p*** **‐values under the alternative hypothesis** ***H***_**1**_ **:** ***γ*** **≠0.** For each value of the rate ratio *f*=*γ*/*ρ*, 100 data sets with sample sizes of *n*=20 and *n*=35 haplotypes, respectively, are independently generated using the MS program [17] with a mutation rate *θ*=1.0/kb and a mean tract length *λ*=0.5 kb. The first five sub‐figures show the Q‐Q plots of the bootstrap *p*‐values (*B*=200) versus a uniform distribution. The last sub‐figure plots the power of the test when using 0.05 as a *p*‐value threshold.

Under the alternative hypothesis *H*_1_:*γ*≠0, different combinations of *ρ* and *γ* are chosen in the simulation, and the ratio of gene conversion to crossover rate *f*=*γ*/*ρ* ranges over 0.5,1.0,2.5,5.0 and 10.0. For each parameter setting, we generate 100 data sets with a mutation rate *θ*=1.0/kb, a mean tract length *λ*=0.5 kb, and sample sizes *n*=20 and *n*=35, respectively. Figure 4 shows the bootstrap estimates of the alternative *p*‐values and the power of the test when setting the *p*‐value threshold to 0.05. As the rate ratio *f*=*γ*/*ρ* or the sample size *n* increases, the alternative *p*‐values tend to decrease towards 0, leading to increased power of detecting gene conversion.

### A real biological application

We apply our testing procedure to SNP data sets from two genes, *su*(*s*) and *su*(*w*^*a*^), located near the telomere of the *X* chromosome of African *Drosophila melanogaster* [20]. The lengths of *su*(*s*) and *su*(*w*^*a*^) loci are about 4.1 kb and 2.5 kb, respectively, and they are about 400 kb apart. The *su*(*s*) locus contains 50 haplotypes and 41 SNPs, and the *su*(*w*^*a*^) locus contains 50 haplotypes and 46 SNPs. The two data sets are further divided into overlapping segments of 20 SNPs each (except for the last segment with 21 SNPs), with 15 SNPs of overlap between two adjacent segments. For each segment, we apply our parametric bootstrap procedure with *B*=500 bootstrap samples. The estimated *p*‐values for the null hypotheses *H*_0_:*γ*=0 and *H*_0_:*ρ*=0 are shown in Tables 4 and 5.

**Table 4:** **Bootstrap** ***p*** **‐values for segments of the** ***su(s)*** **locus in** ***D. melanogaster***

**Segment**	**s1**	**s2**	**s3**	**s4**	**s5**	**All**
Length (kb)	1.8	1.8	1.6	2.4	2.3	4.1
*H*_0_:*γ*=0	0.32	0.66	0.01	0.15	0.54	0.03
*H*_0_:*ρ*=0	0.86	0.46	0.92	0.64	0.36	0.45

**Table 5:** **Bootstrap** ***p*** **‐values for segments of the** ***s*** ***u*** **(** ***w*** ^***a***^ **) locus in** ***D. melanogaster***

**Segment**	**s1**	**s2**	**s3**	**s4**	**s5**	**s6**	**All**
Length (kb)	0.4	1.0	1.1	1.8	1.2	1.5	2.5
*H*_0_:*γ*=0	0.01	0.17	0.31	0.19	0.22	0.30	0.0
*H*_0_:*ρ*=0	0.03	0.71	0.49	0.55	0.88	0.89	0.31

For the *su*(*s*) locus, the *p*‐values against *H*_0_:*ρ*=0 for all the segments (including the whole locus) show no evidence of detecting crossover. However, a small *p*‐value (0.01) against *H*_0_:*γ*=0 is observed for the shortest segment s3, and the overall effect is to provide a strong evidence of gene conversion for the whole locus (*p*‐value = 0.03). This is consistent with the conclusion that gene conversion is likely to play a leading role in shaping the observed polymorphism in this region [20].

A similar pattern of the *p*‐values holds for the *su*(*w*^*a*^) locus, except that the *p*‐values against *H*_0_:*γ*=0 and *H*_0_:*ρ*=0 for the shortest segment s1 are both significant at the 5% level: 0.01 and 0.03, respectively. This could imply that while gene conversion rate is high in this short segment, crossover may not have been greatly suppressed. It could also suggest a higher proportion of gene conversions that are accompanied by crossover events.

## Conclusion

In this work, we have introduced a hypothesis testing procedure that can provide statistical evidence from population genetic data about whether one of the two recombination events is absent. By extensive coalescent simulation studies, we have shown that our parametric bootstrap approach is able to yield accurate estimates of the null *p*‐values that closely follow the theoretical prediction. On the other hand, the bootstrap estimates of the alternative *p*‐values tend to concentrate closer to zero. Our results on real SNP data sets from the *su*(*s*) and *su*(*w*^*a*^) loci of African *D. melanogaster* indicate a strong evidence of detecting gene conversion in short segments of these regions. Moreover, crossover may also play an important role in a short segment of the *su*(*w*^*a*^) locus. We believe that our method provides a necessary complement to the existing procedures of estimating meiotic recombination rates from population genetic data, and expect it to be applied to other data sets.

## Additional files

Additional file 1
**Figure S1.** Histograms of the LRT statistic *Λ*(*H*) under the null hypothesis *H*_0_:*γ*=0 (*n*=20). For each value of the nuisance parameter *ρ*(per kb), 100 data sets with a sample size of *n*=20 are independently generated using the MS program [17] with a mutation rate *θ*=1.0/kb. The 95*%* quantiles of the histograms are: 13.49 (*ρ*=0.5), 8.98 (*ρ*=1.0), 8.56 (*ρ*=2.5), 8.18 (*ρ*=5.0), 9.06 (*ρ*=10.0), and 16.53 (*ρ*=20.0), respectively. The red dashed lines correspond to the density of ${\chi _{2}^{2}}$ distribution.

Additional file 2
**Figure S2.** Bootstrap estimates of the *p*‐values under the null hypothesis *H*_0_:*γ*=0 (*n*=20). For each value of the crossover rate *ρ*(per kb), 100 data sets with a sample size of *n*=20 are independently generated using the MS program [17] with a mutation rate *θ*=1.0/kb. Shown in the figure are the Q‐Q plots of the *p*‐values estimated by *B*=200 parametric bootstrap replications versus a uniform distribution.
