# Supplementary figures and images for: Hypothesis testing of meiotic recombination rates from population genetic data
Source: BMC Genet. 2014 Nov 30;15:122. doi: 10.1186/s12863-014-0122-7 (PMC4267743; doi:10.1186/s12863-014-0122-7)

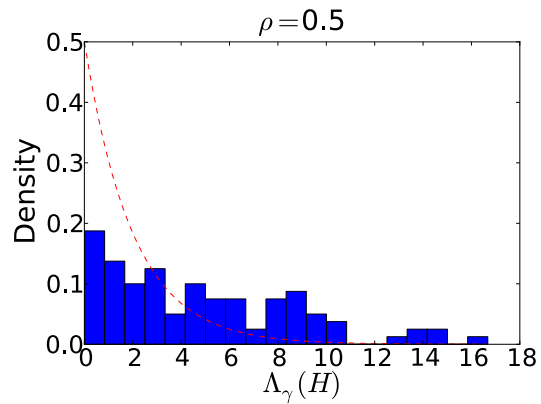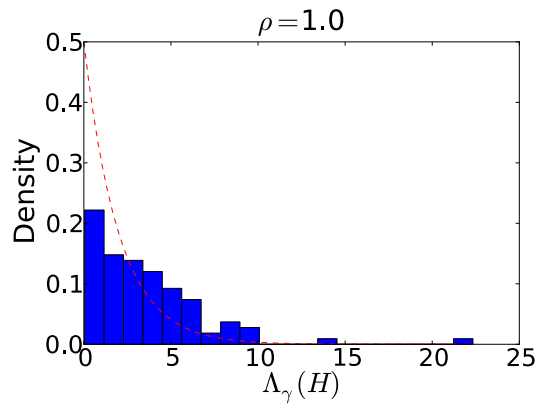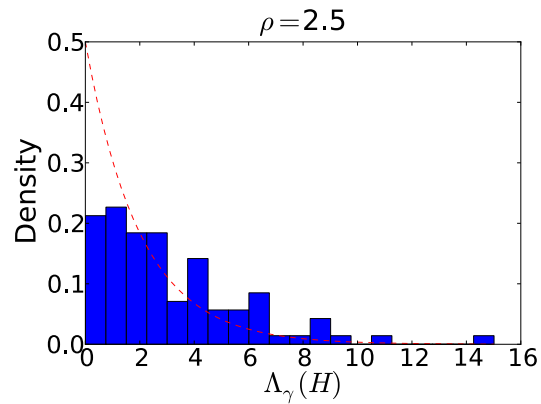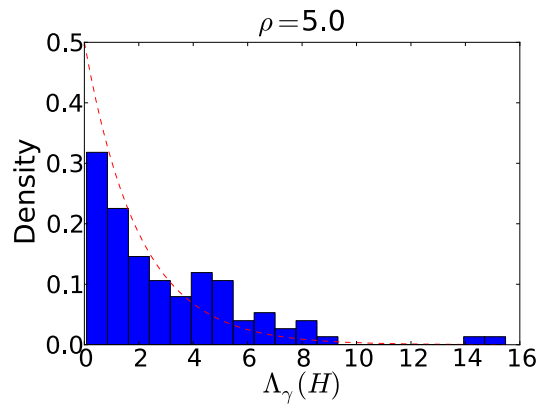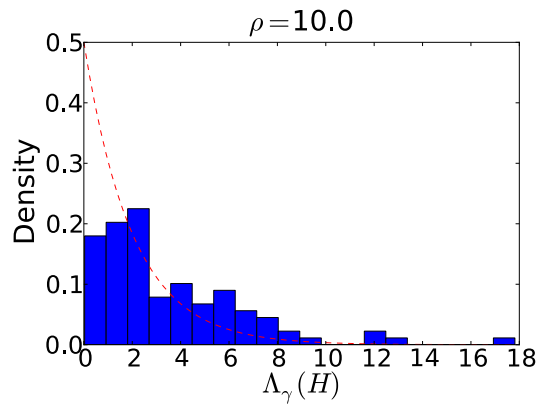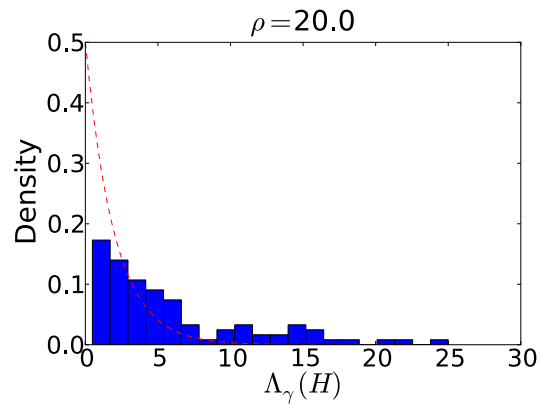

Supplement: Additional file 1 — Figure S1. Histograms of the LRT statistic Λ(H) under the null hypothesis H 0:γ=0 (n=20). For each value of the nuisance parameter ρ(per kb), 100 data sets with a sample size of n=20 are independently generated using the MS program [17] with a mutation rate θ=1.0/kb. The 95% quantiles of the histograms are: 13.49 (ρ=0.5), 8.98 (ρ=1.0), 8.56 (ρ=2.5), 8.18 (ρ=5.0), 9.06 (ρ=10.0), and 16.53 (ρ=20.0), respectively. The red dashed lines correspond to the density of ${\chi _{2}^{2}}$χ22 distribution. [file 12863_2014_122_MOESM1_ESM.pdf]

# Q-Q plots of null p-values

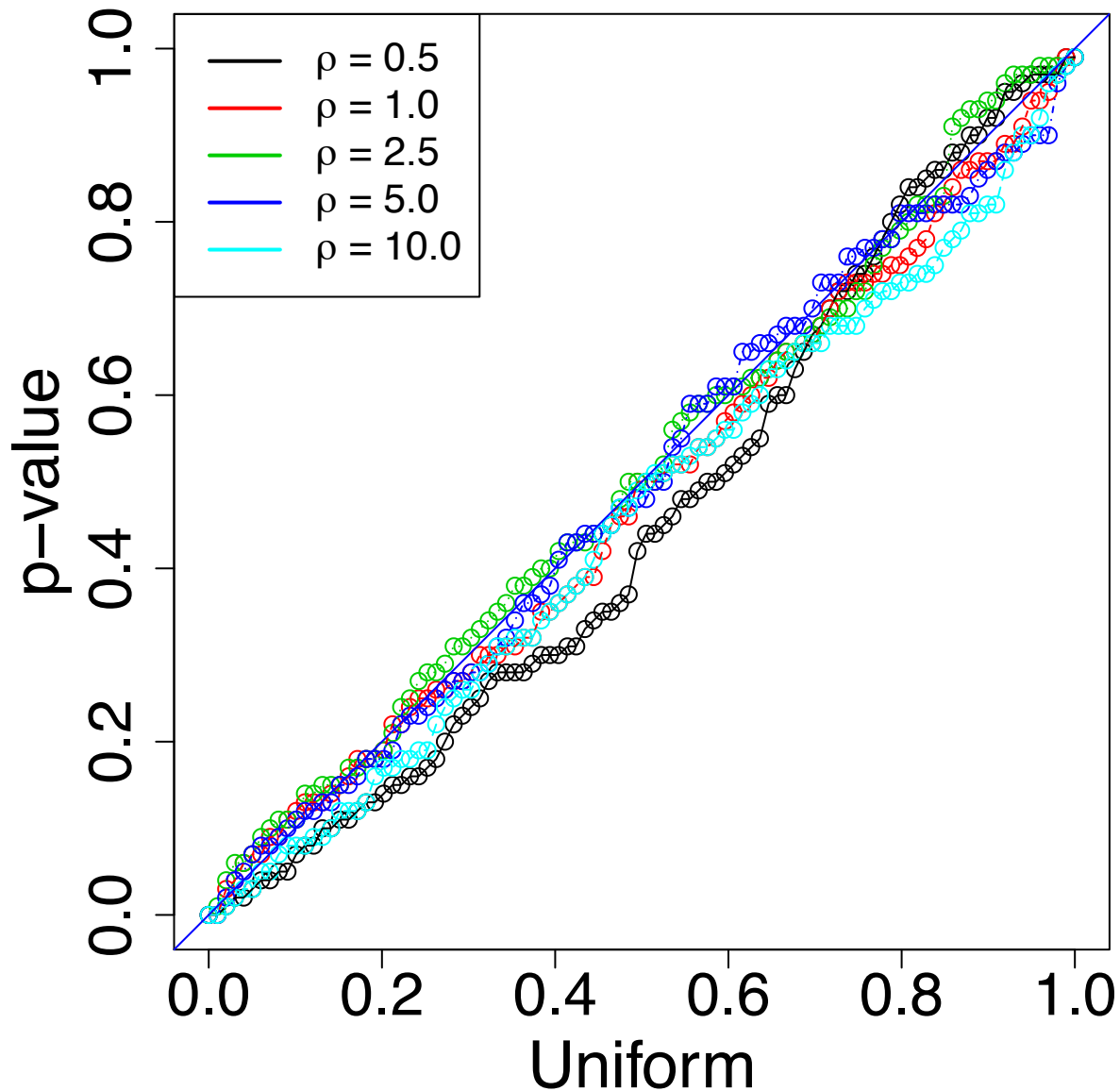

Supplement: Additional file 2 — Figure S2. Bootstrap estimates of the p‐values under the null hypothesis H 0:γ=0 (n=20). For each value of the crossover rate ρ(per kb), 100 data sets with a sample size of n=20 are independently generated using the MS program [17] with a mutation rate θ=1.0/kb. Shown in the figure are the Q‐Q plots of the p‐values estimated by B=200 parametric bootstrap replications versus a uniform distribution. [file 12863_2014_122_MOESM2_ESM.pdf]
